# Social media use and expectations predict COVID-19 vaccine side effects: a prospective longitudinal study

**DOI:** 10.3389/fpsyg.2026.1730585

**Published:** 2026-09-25

**Authors:** Winston Tan, Kirsten Barnes, Kelly S. Clemens, Andrew L. Geers, Evan J. Livesey, Ben Colagiuri

**Affiliations:** 1 School of Psychology, University of Sydney, Sydney, NSW, Australia; 2 Department of Psychology, University of Toledo, Toledo, OH, United States

**Keywords:** COVID-19 vaccine, expectations, nocebo effect, side effects, social media

## Abstract

**Objective:**

Social media use has been implicated in the development of anti-vaccine attitudes. However, its connection with the post-vaccination experience itself remains unclear. The present longitudinal study examined how expectations and psychosocial factors are associated with the relationship between exposure to reports of COVID-19 vaccine side effects on social media and observers’ post-vaccination side effect experience.

**Design and setting:**

Prospective longitudinal study using online surveys.

**Participants and outcome measures:**

Five hundred thirty-eight adults residing in Australia indicated the burden (i.e., number of symptoms and related severity) of COVID-19 vaccine side effects seen reported by other users on social media and their own expectations of experiencing post-vaccination side effects (T1). Perceptions of social media and psychosocial beliefs were also measured. Experience of vaccine-attributed side effects were self-reported after participants received their first (T2; *n* = 299) and second (T3; *n* = 155) vaccine doses.

**Results:**

There was a significant positive association between social media side effect burden exposure and the side effect burden experienced after receiving the first dose. Pre-vaccination side effect expectations formed an indirect association between these variables, but not for the exposure-second dose association. Worry about COVID-19 vaccine side effects, greater side effect severity experienced by known others, and stronger vaccine misconceptions strengthened the first dose indirect association through expectations.

**Conclusion:**

Exposure to user-posted health information on social media was positively associated with post-vaccination side effect experiences and the pre-vaccination expectations associated with this relationship were modulated by several psychosocial factors. Health interventions should consider the potential role of social media-derived symptom information on negative health outcomes.

## Introduction

The COVID-19 pandemic led to an unprecedented global spread of information about vaccines and related post-vaccination side effect risks (Park et al., 2021; Pogue et al., 2020; Prada et al., 2023). A systematic review revealed that three quarters of systemic adverse effects found in the active arms of COVID-19 vaccine trials were also found in the placebo arms, indicating a potential role for the nocebo effect (Haas et al., 2022). The nocebo effect occurs when non-pharmacological properties of a treatment generate negative expectations, subsequently leading to worsened health outcomes. Prospective studies support this, whereby negative pre-vaccination expectations predict experience of COVID-19 vaccine side effects (Geers et al., 2022; Schafer et al., 2023). Interestingly, despite the proliferation of negative information about COVID-19 vaccines on social media, very little research has investigated the extent to which social media is related to the experience of COVID-19 post-vaccination side effects and whether there are additional factors involved in this relationship.

Traditional media (e.g., television news) has a significant influence over the public’s health-related attitudes and behaviors. For example, shortly after the commencement of the Australian COVID-19 vaccination rollout, reports proliferated across media outlets indicating that the AstraZeneca vaccine was associated with blood clotting. This news was followed by a widespread reduction in public intention to be vaccinated, with many individuals citing the blood clotting news as reason for their hesitance (Biddle et al., 2021). In New Zealand, media coverage urged the public to observe for COVID-19 vaccine-related chest pain and changes in heart rate. In the ensuing months, there was a significant increase in reports of these symptoms (MacKrill, 2023). What is interesting here is that the vaccine – and hence the underlying incidence rate – remained the same, but the public’s experience and reporting of those symptoms increased as a result of the media exposure, which is indicative of the nocebo effect. This is not unique to COVID-19. Numerous studies have further demonstrated connections between media coverage and a rise in adverse reaction reports for other medications (Faasse et al., 2012; MacKrill et al., 2020; MacKrill and Petrie, 2018).

Social learning, which occurs when one’s own attitudes, behaviors, and experiences are shaped through observing others (Bandura, 1977) has been proposed as a key mechanism of these media-generated nocebo effects. Because media coverage regularly includes interviews with individuals from the community who share information about their own experiences and health outcomes, viewers are provided with opportunities to obtain second-hand experiential information relevant to their own experiences. Consistent with this, a growing body of experimental research indicates that social learning can drive nocebo effects (see Rooney et al., 2022; Webster et al., 2016 for reviews), even in online contexts (Tan et al., 2023).

Critically, social media offers similar opportunities for people to obtain health information through narrative-style anecdotes and posts about disease and treatment-related health outcomes (Jacques et al., 2022; Madathil et al., 2015). However, unlike traditional media, social media content is largely user-generated and allows for mass scale interaction and exchange of information between individuals. As such, social media may facilitate greater access to social information pertaining to the health-related experiences of others. Concerningly, there is evidence that the use of social media to view social health information can not only reduce psychological wellbeing when the information is negatively valanced, but it can also induce negative expectations for future health experiences (Smailhodzic et al., 2016). As such, social media may serve as fertile grounds for socially learned nocebo effects.

To the best of our knowledge, only two studies have examined the relationship between social media use and COVID-19 vaccine side effects. One study showed that exposure to COVID-19 vaccine side effect posts on social media was positively associated with observers’ first and second dose side effect experiences (Tan et al., 2022). Interestingly, the effect of this exposure was stronger when individuals held greater worry about experiencing post-vaccination side effects, were depressed, anxious, and stressed, or had preferences for social media for vaccine information. However, that study did not include a measure of pre-vaccination expectations, meaning that it could not determine the role of expectations. The second study did, however, identify that vaccine side effects were predicted by the number of social media posts viewed and that these relationships were in fact mediated by side effect expectations. Moreover, the impressions of side effects conveyed by personal acquaintances also predicted pre-vaccination expectations and post-vaccination experiences (Clemens et al., 2023). Preliminary evidence therefore suggests that social media exposure increases risk of post-vaccination nocebo side effects.

While the findings from these two studies are promising, there are some limitations. First, reported side effect outcomes were susceptible to recall bias as first and second dose data in both studies were collected contemporaneously. Though this research suggests a moderating role for psychosocial factors (Tan et al., 2022) and a mediatory role for expectations (Clemens et al., 2023), the interaction between psychosocial factors and expectations has not been investigated. Understanding any interaction here is essential because it is possible that psychosocial factors work to moderate the relationship between social media exposure and negative expectations rather than on the side effect experience directly. This is crucial, because controlling social media content or reducing its use would be largely impractical and therefore not the point at which reduction strategies should intervene. As such, understanding the role of expectations not only provides a means to predict susceptibility to negative health experiences, but the targeting of these expectations may also serve as the only feasible point of intervention to reduce nocebo side effects. Therefore, understanding if there are psychosocial factors that moderate the exposure-expectations relationship could be critical for developing preventative interventions.

To address this, this study aimed to determine whether psychosocial factors modulate the relationship between social media exposure and vaccine side effect expectations, and how these expectations are associated with the first and second dose vaccine side effect experience. It was hypothesized that exposure to user-posted reports of vaccination side effects on social media would be associated with increased pre-vaccination side effect expectations. It was also hypothesized that psychosocial factors would moderate this relationship such that negative expectations would have a stronger indirect association with the relationships between social media exposure and post-vaccination side effect experiences.

## Materials and methods

### Recruitment

Participants were recruited through online panel provider PureProfile (*n* = 506) and social media platforms Facebook, Instagram, and Reddit (*n* = 44) between August and December 2021. To be eligible, participants needed to be unvaccinated for COVID-19 but intending to receive their first dose within 45 days of consenting to participate, fluent in English, and use social media daily for non-work purposes. Participants who met the preregistered exclusion criteria of experiencing extreme symptoms at the time of completing the pre-vaccination survey (>3 SD above the mean; *n* = 12) were removed from analyses. The final pre-vaccination survey sample consisted of unvaccinated adults residing in Australia (*N* = 538; see Table 1 for demographic characteristics). Of this pre-vaccination sample, 55.6% of participants (*n* = 299) responded to the follow-up first dose vaccination survey, with 28.8% (*n* = 155) also completing the second dose vaccination survey. Digital written informed consent was obtained from participants and a debrief statement was provided upon conclusion of the study. Ethical approval was granted by the [masked for anonymised review] Human Ethics Committee [code masked for anonymised review].

**Table 1:** Demographic characteristics of the study sample.

**Variable**	**Mean (*SD*) / *n* (%)**
Age (mean)	40.43 (13.29)
Age ranges	
18–29	112 (20.8%)
30–39	190 (35.3%)
40–49	103 (19.1%)
50–59	75 (13.9%)
60+	58 (10.8%)
Gender	
Male	160 (29.7%)
Female	378 (70.3%)
Primary national/group identity	
Aboriginal or Torres Strait Islander	19 (3.5%)
Australian	419 (77.9%)
New Zealander	14 (2.6%)
Other	84 (15.6%)
Prefer not to say	2 (0.4%)
Highest educational attainment	
Less than high school	57 (10.6%)
High school	119 (22.1%)
Some college but no degree	43 (8.0%)
Graduate diploma or certificate	155 (28.8%)
Bachelor’s degree	114 (21.2%)
Master’s degree	41 (7.6%)
Doctoral degree	5 (0.9%)
Professional degree (JD, MD)	4 (0.7%)
Political view	
Left-leaning	118 (21.9%)
Centre	154 (28.6%)
Right-leaning	74 (13.8%)
Prefer not to say	192 (35.7%)
First-dose vaccine (*n* = 299)	
Pfizer	237 (79.3%)
AstraZeneca	18 (6.0%)
Moderna	43 (14.4%)
N/A	1 (0.3%)
Second-dose vaccine (*n* = 155)	
Pfizer	116 (74.8%)
AstraZeneca	2 (1.3%)
Moderna	26 (16.8%)
N/A	11 (7.1%)

### Materials and measures

Primary measures were assessed using context-specific adaptations of the Generic Assessment of Side Effects questionnaire (GASE; Rief et al., 2011). Each version included 21 items: 18 local and systemic side effects associated with the COVID-19 vaccine (e.g., muscle aches and fatigue) and 3 open-response items where participants could indicate side effects that were not otherwise listed. The social media exposure measure reflected the vaccine-attributed side effects participants had seen reported by other people on social media. The expectation measure assessed vaccine-attributed side effects participants expected to experience in the days after receiving the vaccine. A measure of symptoms being experienced at the time of the pre-vaccination survey was also taken. The first and second dose experience versions were measures of vaccine-attributed side effects participants experienced in the 3 days after receiving each dose, respectively. The fixed symptom content and response format were held constant across versions, while the instructions differed. This approach facilitated content correspondence across the measures. Of note, the resulting scores were intended only as indices of symptom burden rather than a means of constructing new psychometric scales. As such, the context-specific adaptations have not undergone independent validation. Alpha and omega coefficients are reported descriptively for the 18 fixed items.

All items were measured from 1 (*mild*) - 10 (*severe*) with an additional option if they had not seen/expected/experienced the item (coded as 0). Numeric responses to the adapted GASE items could include decimal values and were rounded to the nearest integer before scores were calculated. All primary measures were scored as the sum of the 21 items. This sum represents overall symptom burden (i.e., the joint contribution of symptom breadth and severity) rather than severity alone. For example, a small number of severe symptoms can yield the same sum as many mild symptoms. We therefore also derived the number of distinct symptoms reported (range 0–21) and, among participants reporting at least one symptom, the mean severity of reported symptoms (range 1–10) for descriptive and robustness analyses (see Table 2 for adapted GASE descriptives). General psychological distress was operationalized as the sum of the Depression, Anxiety, and Stress Scale 21 items (DASS-21; Osman et al., 2012), with higher scores indicating greater distress. This overall composite score was selected because the moderator question concerned broad psychological distress; depression, anxiety, and stress subscale item sum scores were examined separately in exploratory sensitivity analyses.

**Table 2:** Descriptives of adapted GASE measures.

**Measure**	***n***	**Sum: *M* (*SD*)**	**Count: *M* (*SD*)**	**Mean reported severity: *M* (*SD*)**
T1 Exposure	538	57.02 (37.43)	10.95 (5.84)	5.10 (1.73); *n* = 495
T1 Expectations	538	41.64 (34.13)	9.84 (5.11)	3.99 (1.87); *n* = 518
T1 Baseline symptoms	538	5.08 (8.61)	1.47 (2.72)	3.74 (2.14); *n* = 248
T2 First dose experience	299	24.35 (23.77)	5.40 (4.26)	4.35 (2.00); *n* = 265
T3 Second dose experience	155	33.34 (26.68)	6.59 (4.54)	4.91 (2.09); *n* = 146

Mean reported severity is defined only for participants endorsing at least one symptom, so its n is smaller.

Full descriptions of other self-report measures are available in Supplementary material. Internal consistency for the 18 adapted fixed GASE items was high for exposure (*α* = 0.933, ω = 0.932), expectations (*α* = 0.946, ω = 0.946), first dose experience (*α* = 0.879, ω = 0.894), and second dose experience (*α* = 0.878, ω = 0.888), and acceptable for baseline symptoms (*α* = 0.729, ω = 0.761). Estimates for the other fixed item scales were the eHealth Literacy Scale (eHEALS; Norman and Skinner, 2006; *α* = 0.934, ω = 0.932), Intolerance of Uncertainty Scale - Short Form (IUS-12; Carleton et al., 2007; *α* = 0.917, ω = 0.918), COVID-19 worry (*α* = 0.954, ω = 0.953), DASS-21 (*α* = 0.960, ω = 0.960), depression subscale (*α* = 0.937, ω = 0.937), anxiety subscale (*α* = 0.878, ω = 0.880), stress subscale (*α* = 0.904, ω = 0.904), and vaccine misconceptions (*α* = 0.839, ω = 0.842).

### Procedure

The present prospective longitudinal study comprised three surveys. After providing consent, participants completed a pre-vaccination survey (T1) that assessed two primary measures: (1) COVID-19 vaccine side effects seen reported by other users on social media (exposure) and (2) their own expectations of experiencing post-vaccination side effects (expectations). This pre-vaccination survey included a baseline measure of symptoms participants were experiencing at the time of taking the survey, measures of psychosocial factors and related vaccine beliefs, and a record of the date participants were receiving their first vaccine dose. Participants then completed the first dose vaccination survey 3–10 days after receiving their first dose (first dose experience; T2) and the second dose vaccination survey 3–10 days after their second dose (second dose experience; T3). The primary outcomes in both post-vaccination surveys measured vaccine-attributed side effects participants experienced in the 3 days after receiving each dose, respectively.

### Statistical analysis

First, a simple linear regression was conducted to examine the association between social media exposure and pre-vaccination side effect expectations. Each of the 18 candidate secondary variables were then tested separately as a moderator of this association. Benjamini-Hochberg adjusted *p* values (*q* values) were calculated across the 18 interaction tests. The depression, anxiety, and stress subscale interactions were examined as a separate exploratory three test family. Mediation analysis was then used to estimate the indirect association between social media exposure and first dose experience through expectations. It should be noted that because exposure and expectations were assessed contemporaneously using identical symptom items and response formats, the direction of the relationship cannot be established in this study. Moderators whose interactions remained supported following Benjamini-Hochberg correction were subsequently examined in separate moderated mediation analyses. Equivalent mediation and moderated mediation analyses were also planned for the second dose experience. Moderation analyses were performed using model 1, mediation analyses using model 4, and moderated mediation analyses using model 7 of PROCESS macro 5.0 (Hayes, 2022). Indirect effects and indices of moderated mediation were estimated using 10,000 bootstrap samples and were considered statistically supported when their 95% bootstrap confidence intervals excluded zero. All other analyses were performed using SPSS Version 32 with an alpha of 0.05.

Potential attrition bias was assessed by comparing T2 and T3 completers and non-completers on the principal T1 study variables, candidate moderators, and key demographic characteristics. Welch independent-samples *t*-tests were used for continuous variables and chi-square tests for categorical variables, with effect sizes expressed as Cohen’s *d* and φ, respectively. These attrition comparisons were descriptive and used unadjusted *p*-values. Pairwise correlations among conceptually overlapping candidate moderators were examined, and tolerance and variance inflation factors from a combined regression model were used to assess multicollinearity. Dose-specific summed symptom burden was compared by gender using Welch independent-samples *t* tests and by age group and vaccine brand using Welch one-way analyses of variance. Age, gender, and vaccine brand were included as covariates in sensitivity analyses of the first and second dose indirect association models. Vaccine brand interaction models were not estimated because some brand-specific cells were sparse, particularly for the second dose. Sensitivity power analyses examined the first dose exposure * moderator interaction component of the second dose moderated mediation models, assuming four predictors, one tested interaction, α = 0.05, and analytic samples of *n* = 155 or *n* = 144 for the ‘experience of known others’ model.

Primary regression and PROCESS analyses used complete cases, with listwise deletion across all variables included in each model, whereas descriptive and bivariate analyses used available cases. As a sensitivity analysis addressing attrition in the primary first dose model, missing first dose symptom burden outcomes were multiply-imputed for the 239 participants who completed T1 but did not provide T2 outcome data. Fifty imputed datasets were generated using the variables in the primary model of exposure, expectations, and baseline symptoms together with the T1 variables that differed between T2 completers and non-completers. The indirect association was estimated within each imputed dataset and pooled across datasets using Rubin’s rules. No other missing values were imputed. Analytic sample sizes are reported for each model.

The study and primary analysis plan were preregistered on AsPredicted https://aspredicted.org/j3fg-fpky.pdf. The preregistration specified analyses of the exposure-expectations association, moderation by the identified secondary variables, baseline-adjusted associations with post-vaccination side effects, and mediation and moderated mediation models involving expectations. All 18 candidate moderators were drawn from the preregistered secondary variables. However, the preregistration described a hierarchical regression framework and did not specify separate one moderator models or adjustment for multiple comparisons. Ethnicity, income, and hours spent reading were not retained because their measurement did not yield sufficiently informative variables for interpretable analyses. Although symptom count scores were included in the preregistered scoring plan, their use in robustness analyses and the calculation of mean severity among reported symptoms were not preregistered. Additional analyses not specified in the preregistration included the Benjamini-Hochberg correction, exploratory DASS-21 subscale analyses, attrition comparisons, demographic and vaccine brand comparisons, sensitivity and power analyses, and the *post hoc* serial mediation model. All data have been made publicly available at Open Science Framework https://osf.io/ga5me.

## Results

### Social media exposure and expectations

A simple linear regression showed a positive association between T1 social media exposure burden and T1 expectation burden, *F*(1, 536) = 372.734, *p* < 0.001, *R*^2^ = 0.410. Exposure burden was associated with higher expectation burden, *B* = 0.584, *SE* = 0.030, 95% CI [0.524, 0.643], *β* = 0.640. The association remained when exposure was operationalized as the number of distinct symptoms seen, *B* = 2.708, *SE* = 0.223, 95% CI [2.269, 3.147], *β* = 0.464, *R*^2^ = 0.215, *p* < 0.001.

Moderation analyses were performed to identify whether any of the secondary factors altered this association between exposure and expectations. After Benjamini-Hochberg correction across the 18 candidate interaction tests, seven moderators were supported: preference for social media as an information source over mainstream media, the strength of that preference, lower mainstream media credibility, greater vaccine side effect worry, more severe side effects reported by known others, stronger vaccine misconceptions, and tertiary education (Table 3). The DASS-21 overall sum score interaction was nominally significant (*p* = 0.0449) but did not survive correction (*q* = 0.1011). Exploratory DASS-21 subscale interactions did not survive correction within their separate three test family: depression *B* = 0.0105, 95% CI [0.0000, 0.0210], *p* = 0.0493, *q* = 0.0746; anxiety *B* = 0.0113, 95% CI [−0.0011, 0.0238], *p* = 0.0746, *q* = 0.0746; and stress *B* = 0.0110, 95% CI [−0.0005, 0.0226], *p* = 0.0610, *q* = 0.0746. Regarding conceptual overlap between variables, there was a maximum pairwise correlation of *r* = 0.720 (media preference category with preference strength); combined model tolerance ranged from 0.418 to 0.743 and VIF from 1.346 to 2.390, providing no evidence of severe multicollinearity. Full reporting can be found in Supplementary material.

**Table 3:** Candidate moderators of the association between T1 social media exposure burden and side effect expectation burden.

**Moderator**	***n***	**Interaction *B* (*SE*)**	**β**	***t***	***p***	***q***	**95% CI**
1. Media preference	538	0.1757 (0.0623)	0.3404	2.8182	0.0050	0.0129	[0.0532, 0.2981]
2. Preference strength	538	0.0032 (0.0010)	0.1974	3.3659	0.0008	0.0032	[0.0013, 0.0051]
3. Preference (statistics and figures)	538	−0.0009 (0.0008)	−0.0716	−1.1650	0.2446	0.3668	[−0.0025, 0.0006]
4. Preference (original post vs. comments)	538	−0.0001 (0.0010)	−0.0098	−0.1502	0.8806	0.8807	[−0.0021, 0.0018]
5. Mainstream media credibility	538	−0.0035 (0.0010)	−0.2665	−3.3386	0.0009	0.0032	[−0.0055, −0.0014]
6. Social media credibility	538	0.0019 (0.0011)	0.1507	1.7048	0.0888	0.1776	[−0.0003, 0.0040]
7. COVID-19 worry	538	−0.0010 (0.0010)	−0.0833	−1.0053	0.3152	0.4364	[−0.0029, 0.0009]
8. COVID-19 vaccine worry	538	0.0065 (0.0009)	0.6662	7.3677	<0.0001	<0.0001	[0.0048, 0.0082]
9. eHealth literacy	538	0.0036 (0.0048)	0.1298	0.7584	0.4485	0.5767	[−0.0057, 0.0130]
10. Intolerance of uncertainty	538	0.0005 (0.0031)	0.0205	0.1523	0.8790	0.8807	[−0.0056, 0.0065]
11. DASS-21 overall sum score	538	0.0042 (0.0021)	0.1482	2.0099	0.0449	0.1011	[0.0001, 0.0082]
12. Experience of known others	479	0.0766 (0.0137)	0.6173	5.5800	<0.0001	<0.0001	[0.0496, 0.1035]
13. Vaccine belief misconceptions	538	0.0011 (0.0002)	0.3120	5.9082	<0.0001	<0.0001	[0.0007, 0.0014]
14. Age	538	−0.0028 (0.0022)	−0.1440	−1.2632	0.2071	0.3388	[−0.0071, 0.0015]
15. Gender	538	0.0166 (0.0652)	0.0199	0.2543	0.7994	0.8807	[−0.1115, 0.1447]
16. Education	538	0.1816 (0.0611)	0.2139	2.9735	0.0031	0.0092	[0.0616, 0.3016]
17. Political view	346	0.0084 (0.0162)	0.0384	0.5207	0.6029	0.7235	[−0.0234, 0.0403]
18. Experience of Flu vaccine side effects	538	−0.1310 (0.0848)	−0.2805	−1.5443	0.1231	0.2216	[−0.2977, 0.0356]

Fifty-nine participants indicated that they did not personally know someone who experienced side effects (Experience of Known Others). One hundred ninety-two participants preferred not to indicate their political view (Political view). *q* is the Benjamini-Hochberg adjusted *p*-value for the 18 test family. Gender was coded 0 = male and 1 = female; education was coded 0 = secondary qualification or lower and 1 = tertiary qualification.

### Attrition of experience measures

The principal T1 study variables, candidate moderators, and key demographic characteristics were compared between follow-up completers and non-completers. The full comparison table is reported in the Supplementary material. Most differences were not statistically supported. At T2, differences with unadjusted *p* < 0.05 were small: non-completers had higher expectation burden (*M* = 45.51 vs. 38.55; *p* = 0.022, *d* = 0.20), less negative media preference strength scores (*M* = −1.14 vs. −6.54; *p* = 0.037, *d* = 0.18), lower statistics and figures preference scores (*M* = −17.08 vs. −8.65; *p* = 0.023, *d* = −0.20), and higher DASS-21 anxiety subscale scores (*M* = 5.40 vs. 4.55; *p* = 0.037, *d* = 0.18). At T3, non-completers were older (*M* = 41.94 vs. 36.70 years; *p* < 0.001, *d* = 0.40) and had lower IUS-12 scores (*M* = 34.63 vs. 37.28; *p* = 0.004, *d* = −0.27). Retention was lower among men than women (21.9% vs. 31.7%, *p* = 0.021, φ = 0.100) and among participants with secondary or lower rather than tertiary education (23.7% vs. 32.3%, *p* = 0.032, φ = 0.093).

Summed symptom burden did not differ by gender after the first dose, Welch *t*(135.33) = −0.38, *p* = 0.703, or second dose, Welch *t*(62.28) = −0.90, *p* = 0.369. Summed burden also did not differ across age groups after the first dose, Welch *F*(4, 119.42) = 0.87, *p* = 0.485, or second dose, Welch *F*(4, 14.23) = 0.55, *p* = 0.706. First dose burden differed by vaccine brand, Welch *F*(2, 36.28) = 4.06, *p* = 0.026 (Pfizer *M* = 22.30; Moderna *M* = 27.23; AstraZeneca *M* = 45.83; AstraZeneca *n* = 18). The second dose brand comparison was not statistically supported, Welch *F*(2, 2.60) = 2.54, *p* = 0.245 (Pfizer *M* = 30.41; Moderna *M* = 47.31; AstraZeneca *M* = 44.00), although this estimate was particularly imprecise because only two AstraZeneca recipients contributed data. Covariate-adjusted sensitivity models are reported below.

### Social media exposure and side effect experiences

A mediation model examined the association of T1 exposure with T2 first dose symptom burden through T1 expectations (*n* = 299; Figure 1). Exposure had a positive total association with first dose burden, *c* = 0.226, 95% CI [0.154, 0.297], *p* < 0.001, *β* = 0.332. Exposure was positively associated with expectations, *a* = 0.500, 95% CI [0.421, 0.578], *β* = 0.582, and expectations were positively associated with first dose burden after adjustment for exposure and baseline symptoms, *b* = 0.302, 95% CI [0.204, 0.401], *β* = 0.382. The direct exposure-first dose association included zero, *c*′ = 0.075, 95% CI [−0.009, 0.158], *p* = 0.080, *β* = 0.110. The indirect effect through expectations was 0.151, boot *SE* = 0.034, 95% bootstrap CI [0.089, 0.225]; the standardized indirect effect was 0.222, 95% bootstrap CI [0.136, 0.317]. In a sensitivity model additionally adjusted for age, gender, and vaccine brand (*n* = 298; one participant without vaccine brand data), the total effect *B* = 0.222, 95% CI [0.153, 0.291]; and indirect effect *B* = 0.134, 95% bootstrap CI [0.079, 0.198] remained supported. Regarding the direct effect *B* = 0.088, 95% CI [0.007, 0.170], although the confidence interval narrowly excluded zero, the effect size was small. Because the corresponding direct association interval included zero in the primary model, this difference should be interpreted cautiously.

**Figure 1:**
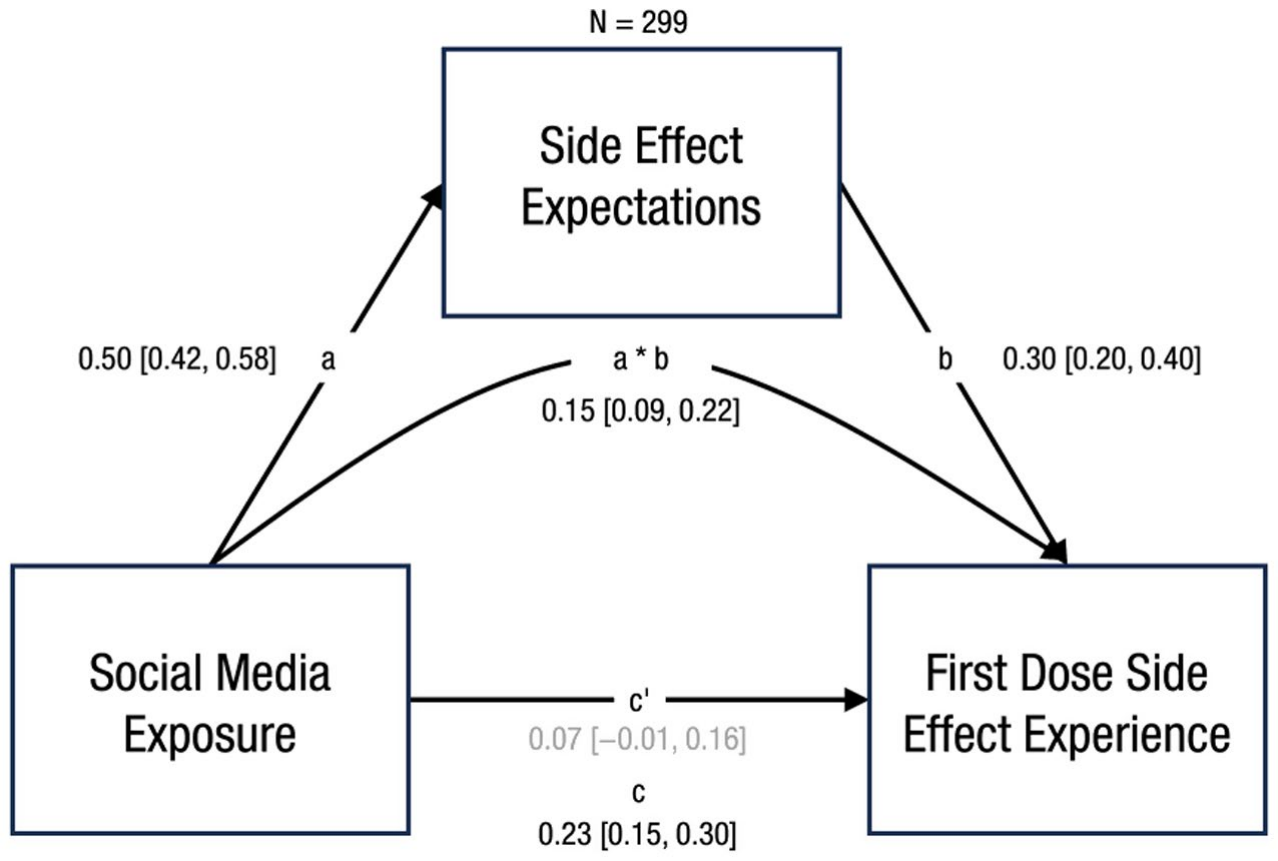
Mediation model examining expectations as a proposed mediator of the association between social media exposure and first dose side effect experience. Values are unstandardized B coefficients with model-based 95% CIs, except the indirect effect, which has a 95% bootstrap CI. Path c is the total association, path c′ is the direct association after adjustment for expectations, and a * b is the statistical indirect effect through expectations. Baseline symptoms were included as a covariate but are omitted from the diagram for clarity. Black and gray values indicate intervals that exclude and include zero, respectively.

Across 50 imputed datasets containing all 538 T1 participants (299 with observed and 239 with imputed first dose outcomes), social media exposure was positively associated with expectations after adjustment for baseline symptoms, *a* = 0.575, 95% CI [0.516, 0.634], *p* < 0.001. In addition to the variables in the primary model, the imputation model included media preference strength, preference for statistics and figures, and DASS-21 anxiety as auxiliary variables because these T1 measures differed between T2 completers and non-completers. Expectations were positively associated with first dose symptom burden after adjustment for exposure and baseline symptoms, *b* = 0.272, 95% CI [0.167, 0.377], *p* < 0.001. The total association was positive, *c* = 0.231, 95% CI [0.158, 0.303], *p* < 0.001, whereas the direct association was not statistically supported, *c*′ = 0.074, 95% CI [−0.004, 0.152], *p* = 0.063. The pooled indirect association through expectations was *B* = 0.156, 95% CI [0.094, 0.219], *p* < 0.001. This pattern was consistent with the primary complete case analysis. As noted in the methods, because both exposure and expectations were measured together at T1, these results, and any following mediation models, should be interpreted as an indirect association rather than as establishing mediation.

The eight first dose moderated mediation models are summarized in Table 4. Seven moderators survived the adjusted T1 interaction screen, and DASS-21 overall score was retained as an exploratory model because its interaction was nominally significant pre-correction. Three indices of moderated mediation had bootstrap intervals excluding zero: vaccine side effect worry, index = 0.0016, 95% bootstrap CI [0.0006, 0.0027]; side effect severity reported by known others, index = 0.0175, 95% bootstrap CI [0.0025, 0.0385]; and vaccine misconceptions, index = 0.0003, 95% bootstrap CI [0.0001, 0.0005]. The indices for social media preference, preference strength, mainstream media credibility, DASS-21 overall score, and education included zero.

**Table 4:** Moderated mediation for social media exposure (X) on first dose experience (Y) with side effect expectations as a mediator (M).

Moderator	*n*	Index	Boot *SE*	95% CI
Social media preference	299	0.0595	0.0344	[−0.0014, 0.1329]
Preference strength	299	0.0009	0.0006	[−0.0001, 0.0023]
Mainstream media credibility	299	−0.0005	0.0007	[−0.0019, 0.0008]
COVID-19 vaccine worry	299	0.0016	0.0005	[0.0006, 0.0027]
DASS-21 overall sum score	299	0.0016	0.0013	[−0.0007, 0.0045]
Experience of known others	267	0.0175	0.0092	[0.0025, 0.0385]
Vaccine misconceptions	299	0.0003	0.0001	[0.0001, 0.0005]
Education	299	0.0235	0.0355	[−0.0401, 0.1003]

Indices are unstandardized indices of moderated mediation with bootstrap 95% CIs. Models included baseline symptoms as a covariate, but values are not presented here for brevity.

Equivalent mediation analyses were conducted with the second dose experience. In the second dose mediation model (*n* = 155; Figure 2), exposure had a positive total association with second dose symptom burden, *c* = 0.161, 95% CI [0.035, 0.286], *p* = 0.013, *β* = 0.204. The exposure-expectation path was positive, *a* = 0.465, 95% CI [0.362, 0.568], *β* = 0.572, but expectations were not uniquely associated with second dose burden, *b* = 0.095, 95% CI [−0.100, 0.290], *p* = 0.336, *β* = 0.098. The direct association included zero, *c*′ = 0.117, 95% CI [−0.038, 0.271], *p* = 0.139, *β* = 0.148. The indirect effect of 0.044 through expectations was not supported, boot *SE* = 0.045, 95% bootstrap CI [−0.051, 0.133]; the completely standardized indirect effect was 0.056, 95% bootstrap CI [−0.062, 0.172]. In a corresponding sensitivity model adjusting for age, gender, and vaccine brand (*n* = 144; 11 participants without vaccine brand data), the total association remained supported, *B* = 0.159, 95% CI [0.023, 0.295], whereas the direct association, *B* = 0.117, 95% CI [−0.049, 0.283], and indirect effect, *B* = 0.042, 95% bootstrap CI [−0.062, 0.136], included zero.

**Figure 2:**
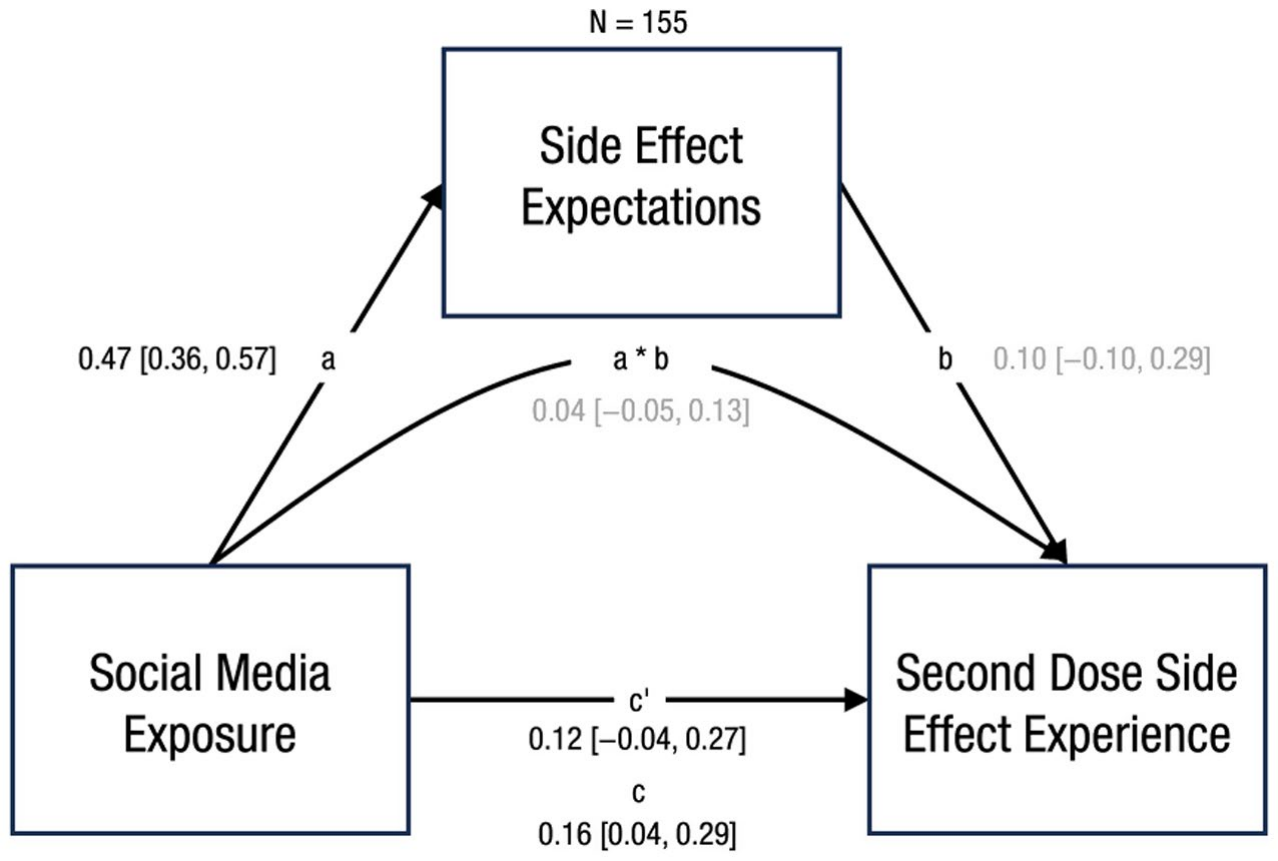
Mediation model examining expectations as a proposed mediator of the association between social media exposure and second dose side effect experience. Values are unstandardized B coefficients with model-based 95% CIs, except the indirect effect, which has a 95% bootstrap CI. Path c is the total association, path c′ is the direct association after adjustment for expectations, and a * b is the statistical indirect effect through expectations. Baseline symptoms were included as a covariate but are omitted from the diagram for clarity. Black and gray values indicate intervals that exclude and include zero, respectively.

All eight second dose indices of moderated mediation included zero, see Supplementary material. Given the reduced second dose sample, regression-based sensitivity power analyses were conducted for the first stage interaction component of the second dose moderated mediation models, assuming four predictors, one tested interaction, and α = 0.05. The sensitivity analyses indicated that the general analytic sample (*n* = 155) provided 80% power to detect an interaction of *f*^2^ = 0.051, whereas power to detect a conventionally small interaction of *f*^2^ = 0.020 was 0.417. For the known others model (*n* = 144), the corresponding values were *f*^2^ = 0.055 and power = 0.392, respectively. Thus, the second dose models had limited power to detect small interaction effects. The model supported a total exposure-second dose association but neither a unique direct association nor an indirect association through T1 expectations. Overall, these findings warrant caution given the substantial attrition and resulting small T3 sample.

### Exploratory analyses

Given the absence of a supported indirect association through expectations in the second dose model, we explored serial mediation as *post hoc* analyses. As personal experience of first dose side effects allows individuals to update their expectations for future doses, the indirect association of expectations may not be with second dose experience directly, but instead through first dose experience (see Figure 3). In this serial model, exposure was associated with expectations, *B* = 0.465, 95% CI [0.362, 0.568], and with first dose burden after adjustment for expectations, *B* = 0.163, 95% CI [0.050, 0.275]. The expectations-first dose path included zero, *B* = 0.136, 95% CI [−0.006, 0.277]. In the second dose equation, expectations had no unique association, *B* = 0.003, 95% CI [−0.169, 0.175], whereas first dose burden was positively associated with second dose burden, *B* = 0.676, 95% CI [0.483, 0.869]. The direct exposure-second dose association included zero, *c*′ = 0.006, 95% CI [−0.133, 0.145]. The total indirect effect was 0.154, 95% bootstrap CI [0.064, 0.248]. The expectations-only indirect effect included zero, Path 1 Index = 0.002, 95% bootstrap CI [−0.081, 0.078]; the first dose only indirect effect excluded zero, Path 2 Index = 0.110, 95% bootstrap CI [0.036, 0.198]; and the serial indirect effect through expectations and first dose burden also excluded zero, Path 3 Index = 0.043, 95% bootstrap CI [0.002, 0.090]. Again, because this model was *post hoc* and the T1 exposure-expectation path was cross-sectional, these findings are exploratory and cannot establish causal mediation.

**Figure 3:**
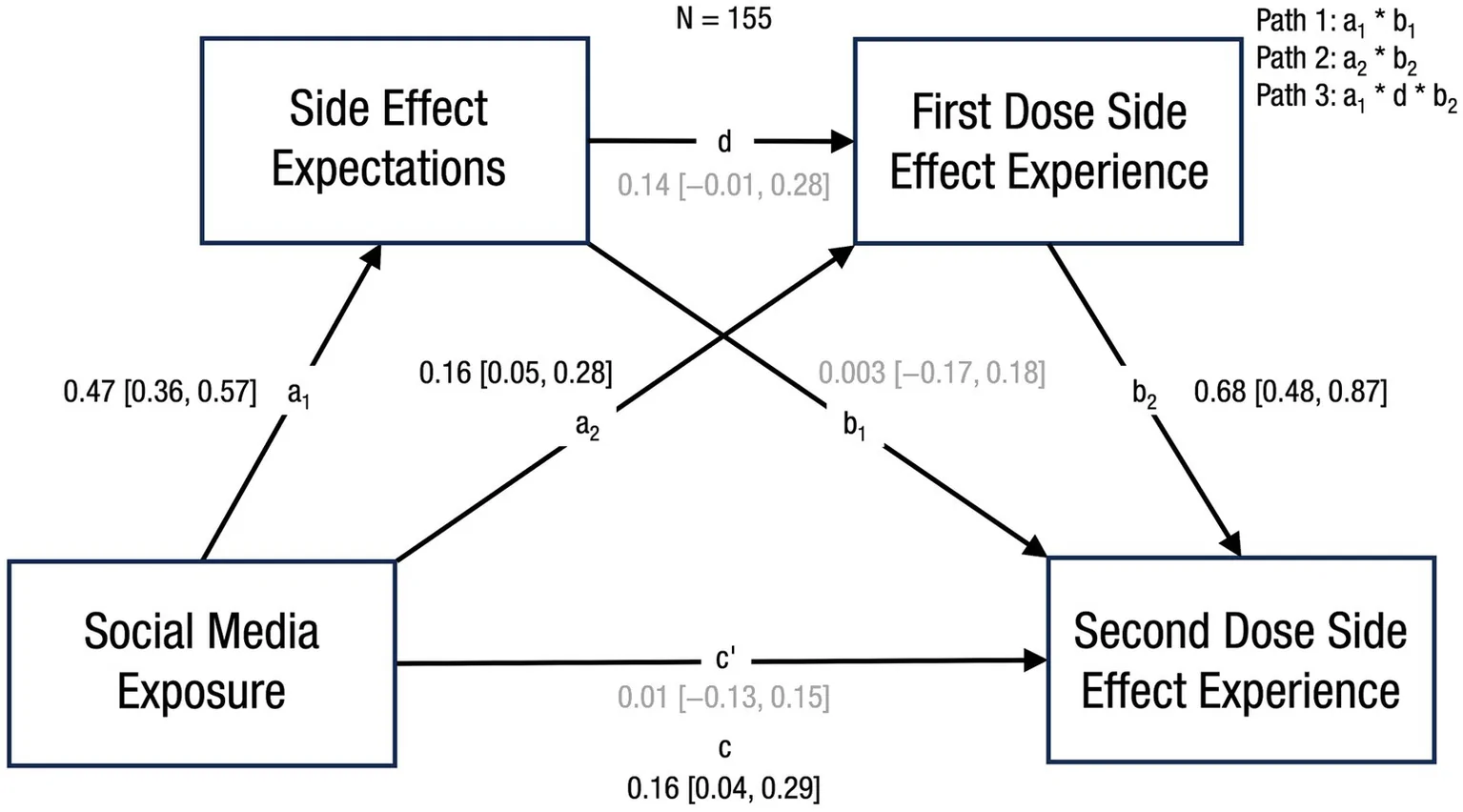
Exploratory serial mediation model examining indirect associations between social media exposure and second dose side effect experience through pre-vaccination expectations and first dose side effect experience. Values are unstandardized B coefficients with 95% CIs; Path 1 is the expectations-only path, Path 2 is the first dose-only path, and Path 3 is the serial path through expectations and first dose burden. The direct path is adjusted for both proposed mediators. Baseline symptoms were included as a covariate but are omitted from the figure for clarity. Black and gray values indicate intervals that exclude and include zero, respectively.

## Discussion

This three-wave prospective study examined whether the severity of COVID-19 vaccine side effects seen reported by others on social media corresponded with participants’ subsequent post-vaccination side effect experience, indirectly through pre-vaccination side effect expectations, and whether psychosocial factors moderated this association. As anticipated, greater exposure to side effect burden on social media showed a positive relationship with greater first dose vaccine side effects experienced. This exposure was also positively associated with negative expectations, which was, in turn, associated with greater first dose side effects experienced, demonstrating an indirect association through expectations. Analyses further demonstrated that this indirect association of expectations was stronger with greater worry about experiencing side effects from the vaccine, greater side effect severity experienced by known others, and stronger vaccine misconceptions. Contrary to what was hypothesized, although social media exposure was positively associated with second dose vaccine side effect burden, the present findings do not support an indirect association through pre-vaccination side effect expectations.

With three in 10 adults indicating that they received a fair amount of information about COVID-19 vaccines from social media early in the vaccine rollout (Hamel et al., 2021) and negative vaccine attitudes affecting the likelihood of developing nocebo-related side effects (Mattarozzi et al., 2023), research into the harmful influence of social media on our health behaviors and experiences is paramount. Preliminary studies have demonstrated that anecdotal information viewed on social media can lead to worse post-vaccination side effect experiences due to the formation of negative side effect expectations (Clemens et al., 2023). Relatedly, several psychosocial factors associated with social media use bolster the positive relationship between social media exposure and the vaccine side effect experience (Tan et al., 2022). The current study extended these findings by demonstrating that these psychosocial factors may modulate the relationship between social media exposure and experience indirectly through negative expectations.

In particular, the present study provides novel evidence that greater beliefs in COVID-19 vaccine misconceptions may worsen our post-vaccination side effect experience by strengthening the association between negative social media health information exposure and negative pre-vaccination expectations. Research suggests that individuals with vaccine-resistant attitudes prefer COVID-19 pandemic-related information from social media platforms where, relative to traditional and authoritative sources, anti-vaccine rhetoric is more rampant (Murphy et al., 2021). Troublingly, those who already hold anti-vaccine attitudes are more strongly influenced by messages and emotional appeals consistent with their ideas (Giese et al., 2020). As such, it is possible that those who reluctantly receive the vaccine may have their expectations more strongly modulated by the negative social information seen on these platforms. More severe side effect experiences of close social others (e.g., friends and family) also strengthened the indirect association of expectations. Potentially knowing people who had more intense side effect experiences may have added to the legitimacy of the personal experiences viewed online and in turn increased the likelihood of individuals updating their side effect expectations to be in line with this anecdotal information, later causing them to experience stronger post-vaccination side effect burden. With that said, due to the contemporaneous measurement of social media exposure and side effect expectations, this interpretation should represent a plausible explanation rather than a definitive account of the observed association.

Nevertheless, these psychosocial factors present potential interventional pathways to mitigate the negative nocebo health outcomes associated with social media exposure. For example, while it is impractical to prevent individuals from learning about the negative experiences of friends and family, as well as others on social media, increasing awareness that knowledge of these second-hand experiences is related to negative side effect expectations, and consequently the experience of side effects, may be a useful strategy to reduce their impact. The current study uniquely highlights that the mitigation of negative attitude-related factors, such as side effect worry and vaccine misconceptions, may serve to diminish the relationship between such exposure and expectations, and consequently reduce the vaccine side effect experience. This is particularly important given that these factors may be much more malleable than exposure to social media itself, both in terms of individuals’ use of social media and the type of content presented on social media. Moreover, the association between exposure and expectations was stronger at lower levels of perceived mainstream media credibility, whereas perceived social media credibility was not supported as a moderator. Although this pattern did not extend to the indirect association with first dose symptom burden, it suggests that skepticism towards mainstream information sources may be relevant to how social media information relates to pre-vaccination expectations.

In addition, educating the public on the ability for the nocebo effect and related social mechanisms to elicit side effect experiences is likely to be fruitful. A handful of studies have found that providing information on the nocebo effect can prevent or reduce the experience of negative health outcomes (Crichton and Petrie, 2015; Karacaoglu et al., 2023; Pan et al., 2019; Wei et al., 2018). These education interventions highlight negative expectations as the primary driver of the nocebo effect. Based on the current findings, future research could explore whether it would be beneficial to additionally explain how psychosocial beliefs may shape the relationship between social learning and negative side effect expectations. This may help individuals recognize the potential negative role of online social information and critically evaluate whether it should inform expectations for their own experiences. Importantly, this aim would be to reduce avoidable expectation-related nocebo outcomes, not to minimize or discourage the reporting of genuine adverse events.

Interestingly, the indirect association between social media exposure and second dose symptom burden through pre-vaccination expectations was not statistically supported. *Post hoc* serial mediation analysis instead indicated that second dose experiences may no longer be related to these pre-vaccination expectations, but instead to the side effects experienced following the first vaccine dose. Given the substantial attrition, reduced T3 sample, and *post hoc* nature of the serial analysis, these findings and following interpretations should be regarded as hypothesis-generating rather than evidence that pre-vaccination expectations ceased to matter. Nonetheless, this outcome is consistent with Smith et al. (2023), who found that the largest predictor of second dose COVID-19 vaccine side effects was side effects experienced following the first dose. However, like the present study, Smith et al. (2023) did not measure expectations after the first dose experience. If individuals update their side effect expectations after personal experience, future studies should include a measure of expectations after the first treatment experience to identify if and how social influence is modulated following first-hand experience. It is possible that if this experience matches that of others, the pre-treatment expectations are strengthened. If they are inconsistent on the other hand, the individual may favor their own experience over others. Pharmacological reactogenicity between types of vaccines may also be a relevant factor to consider. If reactogenicity is stronger for the second Pfizer and Moderna doses, the brands which accounted for most of the vaccines received by participants in the present study, pre-vaccination expectations may be less relevant for the second experience. While adjustments for vaccine brand did not materially alter the first or second dose indirect association estimates, the highly uneven brand distribution, including only two AstraZeneca recipients at the second dose assessment, prevented a reliable examination of whether the expectation pathway differed between vaccine brands.

Some further limitations of the present study and recommendations for future research are worth noting. First, the present sample was recruited at a time when only 30% of the Australian population had not been vaccinated. It is possible that these individuals accumulated significantly more side effect information relative to those first to be vaccinated. In addition, they may represent a more vaccine hesitant population who, as mentioned, are more susceptible to social information and the nocebo effect. This is compounded by the attrition across the three time points. Although most baseline comparisons between completers and non-completers did not differ significantly, follow-up samples may not fully represent the original cohort, particularly at the second dose assessment. Further caution is therefore warranted regarding the generalizability of these findings to other vaccine campaign periods or populations with different levels of vaccine acceptance and social media use. Nonetheless, the positive association between social media exposure and side effect experience has also been found in a sample recruited early in the US vaccination rollout (Clemens et al., 2023), highlighting the relevance of these outcomes throughout a vaccination rollout.

Second, the study involved recruitment of social media users. Future studies should consider including a cohort of non-social media using individuals to identify if there are differences in expectations and side effects experienced between users and non-users. However, we would argue that understanding the effect of social media in social media users is critical and does not diminish the importance of these findings, particularly given the community-based recruitment which allowed for a sample with a wide range of demographics. Third, the Australian booster vaccination program began during recruitment for this study, but data for these doses was not collected. Evidence suggests that intention to receive booster vaccine doses can be modulated psychosocial factors and prior experience (Barnes and Colagiuri, 2022a, 2022b). As such, continuation of the present research into annual vaccine programs (e.g., influenza vaccination) may assist in the improvement of general vaccine side effect experiences. Fourth, as mentioned throughout the paper, the pre-vaccination social media exposure and expectation measures may have affected one other not only due to their contemporaneous measurement (e.g., expectations prior to completion of the T1 survey may have biased what was reported to be seen on social media), but the shared symptom content and response format could further explain the strong positive association between the two. Potential measuring issues are further exacerbated by recall bias, adapted GASE measures not undergoing independent construct validation and, for the post-vaccination experience measures, symptom misattribution. Accordingly, the indirect and conditional associations observed may partly reflect these measurement limitations rather than solely the proposed psychological processes.

In summary, the present study found that greater exposure to reports of vaccine side effects on social media prospectively predicted greater vaccine side effect experience, with an indirect association through pre-vaccination expectations. This indirect association was stronger at higher levels of vaccine side effect worry, greater side effect severity experienced by known others, and stronger belief in vaccine misconceptions. Of these factors, vaccine side effect worry and misconceptions may be more amenable to intervention than exposure to social media information itself, though their modifiability was not examined in this study. Future research should therefore test whether strategies addressing these factors can reduce expectation-related symptom burden related to social media exposure while preserving the appropriate recognition and reporting of genuine adverse events.

## Data Availability

The datasets presented in this study can be found in online repositories. The names of the repository/repositories and accession number(s) can be found at: osf.io/ga5me.
