# Supplementary material for: Social media use and expectations predict COVID-19 vaccine side effects: a prospective longitudinal study
Source: Front Psychol. 2026 Sep 25;17:1730585. doi: 10.3389/fpsyg.2026.1730585 (PMC13650080; doi:10.3389/fpsyg.2026.1730585)
Supplement: Supplementary file 1 [file Data_Sheet_1.docx]

**Supplementary Material**

**Adapted Generic Assessment of Side Effects Item Structure:**

[1] Pain at the injection site

[2] Redness at the injection site

[3] Swelling at the injection site

[4] Itching at the injection site

[5] Fever

[6] Chills (shivering and feeling cold)

[7] Headache

[8] Muscle ache/pain

[9] Joint ache/pain

[10] Fatigue/tiredness

[11] Fainting/loss of consciousness

[12] Convulsions or seizures

[13] Skin rash (not at injection site)

[14] Nausea

[15] Vomiting

[16] Diarrhoea

[17] Abdominal pain

[18] Blood clots

[19] Other side effect 1 (paired free-text description)

[20] Other side effect 2 (paired free-text description)

[21] Other side effect 3 (paired free-text description)

**Table S1.** Secondary variable descriptions

| **Variable** | **Item** | **Scoring** | **Interpretation** |
| --- | --- | --- | --- |
| Social Media Preference | Participants indicated whether they preferred mainstream or social media when seeking information related to the experience of COVID-19 vaccine side effects between 1 – Mainstream media and 2 – Social media (forced choice) | | Higher score = preference for social media |
| Preference Strength | Participants indicated their strength of preference for mainstream or social media from -50 (Mainstream media) to 50 (Social media) | | Higher score = greater preference for social media |
| Preference for Statistics and Figures over Personal Stories (A)  Preference for  Original Posts over comments or replies (B) | Participants rated how valuable they found the following pieces of information when reading social media posts about COVID-19 vaccine side effects from 0 (Not at all valuable) – 100 (Extremely valuable):   1) Statistics and figures posted by the original poster 2) Statistics and figures presented by other posters in the comments or replies 3) Personal stories posted by the original poster 4) Personal stories posted by other posters in the comments or replies | A = (1+2) - (3+4)  B = (1+3) - (2+4) | Higher score = greater preference for statistics and figures over personal stories  Higher score = greater preference for original posts over comments or replies |
| Mainstream Media Credibility | Participants rated how credible they believed mainstream media to be in providing accurate information related to the experience of COVID-19 vaccine side effects from 0 (Not at all credible) – 100 (Very credible) | | Higher score = greater belief in mainstream media as a credible source |
| Social Media Credibility | Participants rated how credible they believed social media to be in providing accurate information related to the experience of COVID-19 vaccine side effects from 0 (Not at all credible) – 100 (Very credible) | | Higher score = greater belief in social media as a credible source |
| COVID-19 Worry | Measured using the COVID-19 worry scale (Ahmed et al., 2020) | Mean average of 7 items, 0-100 | Higher score = greater worry about COVID-19 |
| COVID-19 Vaccine Worry | Participants indicated how worried they were about experiencing side effects from the COVID-19 vaccine from 0 (Not at all worried) – 100 (Extremely worried) | | Higher score = greater worry about experiencing side effects |
| eHealth Literacy | eHealth Literacy Scale (Norman & Skinner, 2006) | Sum of 8 items, 8-40 | Higher score = greater eHealth literacy |
| Intolerance of Uncertainty | Intolerance of Uncertainty Scale – Short Form (Carleton et al., 2007) | Sum of 12 items, 12-60 | Higher score = greater intolerance of uncertainty |
| Depression Anxiety Stress Scale Score (DASS-21) | Depression, Anxiety, and Stress Scale 21 (Osman et al., 2012) | Sum of the 21 items, 0-63. Subscales calculated as the sum of respective items, 0-21. | Higher score = greater overall depression, anxiety, & stress-related symptoms |
| Experience of Known Others | Participants indicated how severe the COVID-19 vaccine side effects experienced by those personally known to them were from 0 (No side effects) – 10 (Severe side effects) | | Higher score = more severe side effect experience of personally known others |
| Vaccine Belief Misconceptions | Participants rated the extent to which they believed in eight common vaccine misconceptions (e.g., the COVID-19 vaccines can affect a person’s fertility) from -50 (Definitely untrue) – 50 (Definitely true) | Sum of 8 items, -400-400 | Higher score = greater belief in vaccine misconceptions |
| Political View | Participants indicated where on the left-right political spectrum they identified with from -5 (left) to 0 (centre) to 5 (right). There was also an additional option where participants could indicate if they preferred not to say. | | -5 to -1 = Left-leaning  0 = Centre  1 to 5 = Right-leaning |
| Experience of flu vaccine side effects | Participants indicated whether they had experienced side effects from flu vaccination in the past between 1 – Yes and 2 - No | |  |

| Variable | Non-completer  *n* | Non-completer  *M* (*SD*) | Completer  *n* | Completer  *M* (*SD*) | Welch *t* | *df* | *p* | *d* |
| --- | --- | --- | --- | --- | --- | --- | --- | --- |
| Age (years) | 239 | 39.26 (12.82) | 299 | 41.37 (13.61) | -1.85 | 521.8 | .065 | -.16 |
| Exposure Burden (sum) | 239 | 59.44 (40.23) | 299 | 55.08 (34.99) | 1.32 | 474.3 | .187 | .12 |
| Exposure Symptom count | 239 | 11.16 (6.02) | 299 | 10.78 (5.70) | .76 | 497.6 | .448 | .07 |
| Expectation Burden (sum) | 239 | 45.51 (38.34) | 299 | 38.55 (30.05) | 2.30 | 443.6 | .022 | .20 |
| Expectation Symptom count | 239 | 10.13 (5.40) | 299 | 9.61 (4.87) | 1.15 | 484.7 | .250 | .10 |
| Baseline Symptom Burden | 239 | 5.23 (8.82) | 299 | 4.96 (8.45) | .36 | 500.2 | .722 | .03 |
| Media Preference Strength | 239 | -1.14 (30.03) | 299 | -6.54 (29.36) | 2.09 | 505.1 | .037 | .18 |
| Statistics/Figures Preference | 239 | -17.08 (44.87) | 299 | -8.65 (39.36) | -2.29 | 476.7 | .023 | -.20 |
| Original Post Preference | 239 | 14.48 (33.39) | 299 | 18.28 (33.84) | -1.30 | 513.1 | .192 | -.11 |
| Mainstream Media Credibility | 239 | 50.87 (28.52) | 299 | 53.26 (26.20) | -1.00 | 489.5 | .318 | -.09 |
| Social Media Credibility | 239 | 46.77 (24.71) | 299 | 43.60 (26.62) | 1.43 | 524.1 | .153 | .12 |
| COVID-19 Worry | 239 | 53.11 (29.89) | 299 | 51.60 (29.70) | .59 | 508.8 | .558 | .05 |
| COVID-19 Vaccine Worry | 239 | 56.28 (30.98) | 299 | 53.84 (30.67) | .91 | 508.0 | .362 | .08 |
| eHealth Literacy | 239 | 29.82 (6.57) | 299 | 29.73 (5.77) | .16 | 477.1 | .872 | .01 |
| Intolerance of Uncertainty | 239 | 34.76 (10.21) | 299 | 35.90 (9.89) | -1.30 | 502.9 | .193 | -.11 |
| DASS-21 Overall Sum Score | 239 | 19.88 (14.76) | 299 | 17.85 (13.53) | 1.64 | 488.9 | .101 | .14 |
| DASS-21 Depression | 239 | 6.88 (5.87) | 299 | 6.30 (5.46) | 1.17 | 492.8 | .242 | .10 |
| DASS-21 Anxiety | 239 | 5.40 (4.93) | 299 | 4.55 (4.42) | 2.09 | 482.7 | .037 | .18 |
| DASS-21 Stress | 239 | 7.60 (5.11) | 299 | 7.00 (4.77) | 1.38 | 494.0 | .167 | .12 |
| Experience of Known Others | 212 | 4.37 (2.16) | 267 | 4.09 (2.03) | 1.43 | 439.6 | .152 | .13 |
| Vaccine Belief Misconceptions | 239 | -77.85 (145.89) | 299 | -102.10 (142.78) | 1.93 | 505.4 | .054 | .17 |
| Political View | 148 | -0.48 (2.21) | 198 | -0.29 (2.36) | -.75 | 326.9 | .451 | -.08 |

**Table S2.** T1 continuous characteristics by valid first dose (T2) sample inclusion

**Table S3.** T1 continuous characteristics by valid second dose (T3) sample inclusion

| Variable | Non-completer  *n* | Non-completer  *M* (*SD*) | Completer  *n* | Completer  *M* (*SD*) | Welch *t* | *df* | *p* | *d* |
| --- | --- | --- | --- | --- | --- | --- | --- | --- |
| Age (years) | 383 | 41.94 (14.12) | 155 | 36.70 (10.09) | 4.83 | 394.9 | < .001 | .40 |
| Exposure Burden (sum) | 383 | 58.23 (38.79) | 155 | 54.03 (33.80) | 1.25 | 324.7 | .212 | .11 |
| Exposure Symptom count | 383 | 11.17 (5.81) | 155 | 10.41 (5.90) | 1.36 | 281.4 | .175 | .13 |
| Expectation Burden (sum) | 383 | 42.86 (36.43) | 155 | 38.64 (27.49) | 1.46 | 374.5 | .145 | .12 |
| Expectation Symptom count | 383 | 9.82 (5.29) | 155 | 9.89 (4.68) | -.16 | 319.9 | .875 | -.01 |
| Baseline Symptom Burden | 383 | 5.00 (8.48) | 155 | 5.29 (8.93) | -.35 | 272.5 | .727 | -.03 |
| Media Preference Strength | 383 | -3.18 (30.11) | 155 | -6.52 (28.80) | 1.20 | 297.0 | .230 | .11 |
| Statistics/Figures Preference | 383 | -13.07 (43.25) | 155 | -10.74 (39.08) | -.61 | 313.5 | .544 | -.06 |
| Original Post Preference | 383 | 16.17 (34.19) | 155 | 17.64 (32.40) | -.47 | 299.5 | .640 | -.04 |
| Mainstream Media Credibility | 383 | 51.35 (27.86) | 155 | 54.29 (25.66) | -1.17 | 307.8 | .241 | -.11 |
| Social Media Credibility | 383 | 46.00 (25.81) | 155 | 42.55 (25.74) | 1.41 | 285.7 | .160 | .13 |
| COVID-19 Worry | 383 | 51.83 (29.78) | 155 | 53.35 (29.80) | -.54 | 284.8 | .592 | -.05 |
| COVID-19 Vaccine Worry | 383 | 55.33 (30.99) | 155 | 53.93 (30.42) | .48 | 290.0 | .630 | .05 |
| eHealth Literacy | 383 | 29.80 (6.31) | 155 | 29.68 (5.68) | .23 | 314.3 | .821 | .02 |
| Intolerance of Uncertainty | 383 | 34.63 (10.30) | 155 | 37.28 (9.14) | -2.94 | 318.7 | .004 | -.27 |
| DASS-21 Overall Sum Score | 383 | 18.76 (14.27) | 155 | 18.74 (13.78) | .01 | 294.2 | .991 | .00 |
| DASS-21 Depression | 383 | 6.52 (5.72) | 155 | 6.66 (5.47) | -.27 | 297.2 | .784 | -.03 |
| DASS-21 Anxiety | 383 | 5.01 (4.70) | 155 | 4.72 (4.58) | .66 | 291.8 | .508 | .06 |
| DASS-21 Stress | 383 | 7.23 (5.00) | 155 | 7.36 (4.76) | -.29 | 298.0 | .775 | -.03 |
| Experience of Known Others | 335 | 4.27 (2.15) | 144 | 4.08 (1.95) | .95 | 297.1 | .343 | .09 |
| Vaccine Belief Misconceptions | 383 | -83.89 (143.88) | 155 | -109.71 (144.97) | 1.87 | 283.1 | .062 | .18 |
| Political View | 244 | -0.35 (2.34) | 102 | -0.42 (2.22) | .26 | 198.6 | .795 | .03 |

**Table S4.** Categorical T1 characteristics and retention in the valid T2 first dose and T3 second dose samples

| Characteristic | Category | T2 completers *n*/*N* (%) | T2 χ²(1) | T2 *p* | T2 φ | T3 completers *n*/*N* (%) | T3 χ²(1) | T3 *p* | T3 φ |
| --- | --- | --- | --- | --- | --- | --- | --- | --- | --- |
| Gender | Men | 82/160 (51.2%) | 1.73 | .189 | 0.057 | 35/160 (21.9%) | 5.34 | .021 | 0.100 |
|  | Women | 217/378 (57.4%) |  |  |  | 120/378 (31.7%) |  |  |  |
| Education | Secondary qualification or lower | 112/219 (51.1%) | 2.94 | .086 | 0.074 | 52/219 (23.7%) | 4.62 | .032 | 0.093 |
|  | Tertiary qualification | 187/319 (58.6%) |  |  |  | 103/319 (32.3%) |  |  |  |
| Media Preference | Mainstream media | 185/320 (57.8%) | 1.60 | .206 | 0.055 | 96/320 (30.0%) | 0.54 | .460 | 0.032 |
|  | Social media | 114/218 (52.3%) |  |  |  | 59/218 (27.1%) |  |  |  |
| Prior flu vaccine side effects | Yes | 41/80 (51.2%) | 0.71 | .399 | 0.036 | 25/80 (31.2%) | 0.27 | .602 | 0.023 |
|  | No | 258/458 (56.3%) |  |  |  | 130/458 (28.4%) |  |  |  |

**Table S5.** Vaccine-attributed symptom burden by demographic characteristics and vaccine brand

| Outcome | Characteristic | Group | *n* | *M* (*SD*) | Comparison |
| --- | --- | --- | --- | --- | --- |
| First dose (T2) | Gender | Men | 82 | 23.46 (25.35) | Welch *t*(135.3) = -0.38, *p* = .703, *d* = -0.05 |
|  |  | Women | 217 | 24.69 (23.20) |  |
| First dose (T2) | Age group | 18-29 | 57 | 28.32 (23.94) | Welch *F*(4, 119.4) = 0.87, *p* = .485 |
|  |  | 30-39 | 103 | 24.21 (23.52) |  |
|  |  | 40-49 | 57 | 20.74 (19.67) |  |
|  |  | 50-59 | 47 | 25.06 (26.29) |  |
|  |  | 60+ | 35 | 23.26 (26.98) |  |
| First dose (T2) | Vaccine brand | Pfizer | 237 | 22.30 (22.00) | Welch *F*(2, 36.3) = 4.06, *p* = .026 |
|  |  | Moderna | 43 | 27.23 (21.66) |  |
|  |  | AstraZeneca | 18 | 45.83 (37.74) |  |
| Second dose (T3) | Gender | Men | 35 | 30.00 (23.99) | Welch *t*(62.3) = -0.90, *p* = .369, *d* = -0.16 |
|  |  | Women | 120 | 34.31 (27.43) |  |
| Second dose (T3) | Age group | 18-29 | 40 | 38.70 (29.13) | Welch *F*(4, 14.2) = 0.55, *p* = .706 |
|  |  | 30-39 | 60 | 32.05 (27.42) |  |
|  |  | 40-49 | 31 | 31.42 (25.00) |  |
|  |  | 50-59 | 21 | 29.00 (21.83) |  |
|  |  | 60+ | 3 | 37.67 (31.97) |  |
| Second dose (T3) | Vaccine brand | Pfizer | 116 | 30.41 (24.53) | Welch *F*(2, 2.6) = 2.54, *p* = .245 |
|  |  | Moderna | 26 | 47.31 (32.25) |  |
|  |  | AstraZeneca | 2 | 44.00 (62.23) |  |

**Table S6.** Correlations among candidate moderators

| Variable | 1 | 2 | 3 | 4 | 5 | 6 | 7 | 8 | 9 | 10 |
| --- | --- | --- | --- | --- | --- | --- | --- | --- | --- | --- |
| 1. Media Preference | — |  |  |  |  |  |  |  |  |  |
| 2. Preference Strength | 0.720 | — |  |  |  |  |  |  |  |  |
| 3. Mainstream Media Credibility | -0.309 | -0.316 | — |  |  |  |  |  |  |  |
| 4. Social Media Credibility | 0.343 | 0.403 | 0.153 | — |  |  |  |  |  |  |
| 5. COVID-19 Worry | 0.007 | -0.079 | 0.242 | 0.118 | — |  |  |  |  |  |
| 6. COVID-19 Vaccine Worry | 0.203 | 0.183 | -0.111 | 0.242 | 0.394 | — |  |  |  |  |
| 7. Intolerance of Uncertainty | 0.023 | 0.006 | 0.190 | 0.122 | 0.332 | 0.310 | — |  |  |  |
| 8. DASS-21 Overall Sum Score | 0.070 | 0.075 | 0.017 | 0.045 | 0.190 | 0.121 | 0.542 | — |  |  |
| 9. Experience of Known Others | 0.098 | 0.134 | -0.110 | 0.187 | 0.070 | 0.397 | 0.057 | 0.031 | — |  |
| 10. Vaccine Belief Misconceptions | 0.218 | 0.265 | -0.205 | 0.309 | 0.026 | 0.426 | 0.170 | 0.175 | 0.282 | — |

**Table S7.** Tolerance and variance inflation factors (VIF) for the exposure and psychosocial predictors

| Predictor | Tolerance | VIF |
| --- | --- | --- |
| Exposure Burden | 0.649 | 1.541 |
| Media Preference | 0.452 | 2.212 |
| Preference Strength | 0.418 | 2.390 |
| Mainstream Media Credibility | 0.683 | 1.463 |
| Social Media Credibility | 0.653 | 1.532 |
| COVID-19 Worry | 0.743 | 1.346 |
| COVID-19 Vaccine Worry | 0.565 | 1.771 |
| Intolerance of Uncertainty | 0.616 | 1.623 |
| DASS-21 Overall Sum Score | 0.695 | 1.439 |
| Experience of Known Others | 0.677 | 1.476 |
| Vaccine Belief Misconceptions | 0.713 | 1.402 |

**Table S8.** Indices of moderated mediation for second dose symptom burden

| Moderator | *n* | Index | Bootstrap *SE* | 95% bootstrap CI |
| --- | --- | --- | --- | --- |
| Media Preference | 155 | 0.0109 | 0.0226 | [−0.0155, 0.0743] |
| Preference Strength | 155 | 0.0000 | 0.0003 | [−0.0005, 0.0007] |
| Mainstream Media Credibility | 155 | −0.0001 | 0.0004 | [−0.0010, 0.0005] |
| COVID-19 Vaccine Worry | 155 | 0.0007 | 0.0007 | [−0.0008, 0.0021] |
| DASS-21 Overall Sum Score | 155 | 0.0006 | 0.0009 | [−0.0006, 0.0028] |
| Experience of Known Others | 144 | 0.0082 | 0.0120 | [−0.0160, 0.0331] |
| Vaccine Belief Misconceptions | 155 | 0.0001 | 0.0001 | [−0.0001, 0.0003] |
| Education | 155 | −0.0041 | 0.0211 | [−0.0622, 0.0254] |

*Note.* PROCESS model 7 with baseline symptom burden as a covariate and 10,000 bootstrap samples. Four decimal places are reported so small indices remain evaluable. Every confidence interval included zero.

**Table S9.** Primary and covariate-adjusted mediation models

| Outcome | Model | *n* | Total effect *B* [95% CI] | Direct effect *B* [95% CI] | Indirect effect *B* [95% boot CI] | Standardized indirect β [95% boot CI] |
| --- | --- | --- | --- | --- | --- | --- |
| First dose | Primary | 299 | 0.2257 [0.1543, 0.2971] | 0.0746 [−0.0089, 0.1581] | 0.1511 [0.0891, 0.2249] | 0.2224 [0.1361, 0.3171] |
| First dose | Age-, gender-, and brand-adjusted | 298 | 0.2220 [0.1530, 0.2910] | 0.0884 [0.0068, 0.1700] | 0.1336 [0.0790, 0.1984] | 0.1968 [0.1199, 0.2834] |
| Second dose | Primary | 155 | 0.1607 [0.0351, 0.2862] | 0.1165 [−0.0384, 0.2713] | 0.0442 [−0.0505, 0.1325] | 0.0560 [−0.0617, 0.1723] |
| Second dose | Age-, gender-, and brand-adjusted | 144 | 0.1591 [0.0232, 0.2951] | 0.1171 [−0.0490, 0.2831] | 0.0421 [−0.0620, 0.1355] | 0.0508 [−0.0727, 0.1673] |
